# Phage Therapy Enhanced by Using Engineered Bacteriophages: A Powerful Antibacterial Tool to Address the Dilemma Posed by Multidrug-Resistant Bacterial Infections

**DOI:** 10.3390/ijms27167103

**Published:** 2026-08-08

**Authors:** Xuanliang Wang, Haolin Zhou, Theam Soon Lim, Grzegorz Węgrzyn

**Affiliations:** 1 College of Life Science and Technology, Huazhong University of Science and Technology, Wuhan 430074, China; xlwang2026@126.com (X.W.); hlzhou2626@163.com (H.Z.); 2 Institute for Research in Molecular Medicine, Universiti Sains Malaysia, Pulau Pinang 11800, Malaysia; 3 Laboratory of Bacteriophage Biology and Biotechnology, Department of Molecular Biology, Faculty of Biology, University of Gdansk, Wita Stwosza 59, 80-308 Gdansk, Poland

**Keywords:** phage therapy, antimicrobial resistance, synthetic biology, engineered bacteriophages, CRISPR-Cas, host range expansion, biofilm degradation, clinical translation

## Abstract

The continuous slowdown in the research and development of new antibiotics and antibiotic overuse have turned the problem of antibacterial resistance into a global public health crisis. As a very promising alternative to multi-drug-resistant bacterial infection, phage therapy is receiving renewed attention. However, the inherent biological limitations of natural phages restrict their extensive clinical application. This review examines how synthetic biology can be harnessed to transform phages and to build the next generation of antibacterial therapies. We outline the main advantages of natural phages, including high host specificity, self-amplification, bactericidal activity and the ability to degrade biofilms. We also point out the bottlenecks of clinical applications of bacteriophages, such as narrow host range, rapid removal in the body and potential genetic safety risks. Moreover, we elaborate on the core synthetic biological tools used to overcome the above limitations, including CRISPR-Cas gene editing, receptor-binding protein reprogramming, functional load delivery and immunogenic regulation, and summarize the recent clinical progress and personalized treatment process. The increasing clinical evidence shows that synthetic biology can effectively overcome the inherent defects of natural bacteriophages, confirming the safety and initial efficacy of bacteriophage therapy. Engineered phages provide a practical strategy to meet the antimicrobial resistance challenge. Clinical applications of such phages will mainly depend on progress in production standardization, regulatory framework construction and scientific and reasonable joint treatment program development.

## 1. Introduction

The discovery and clinical application of antibiotics is one of the most transformative achievements of modern medicine. It has transformed once deadly bacterial infections into controllable diseases and extended global life expectancy by decades [1,2]. However, nearly a century after Alexander Fleming’s isolation of penicillin, the indiscriminate use and overprescription of antibiotics have precipitated an unprecedented crisis of antimicrobial resistance (AMR) [3]. The report, Global Burden of Bacterial Antimicrobial Resistance in 2019: a Systematic Analysis, estimates that 1.27 million deaths are directly attributable to drug-resistant infections annually, with an additional 4.95 million fatalities linked to AMR-related complications [4]. ESKAPE pathogens (*Enterococcus faecium*, *Staphylococcus aureus*, *Klebsiella pneumoniae*, *Acinetobacter baumannii*, *Pseudomonas aeruginosa*, and *Enterobacter* species) account for the majority of hospital-acquired drug-resistant infections, posing an existential threat to modern healthcare systems [5]. Compounding this crisis, the pipeline for novel small-molecule antibiotics has stagnated dramatically, leaving humanity on the brink of a post-antibiotic era where even minor infections could become fatal [6].

Against this bleak backdrop, phage therapy, the therapeutic use of bacteriophages, that is, viruses that exclusively infect and lyse bacteria, has experienced a remarkable global renaissance. Bacteriophages were independently discovered by Frederick Twort in 1915 and Félix d’Hérelle in 1917, and were already employed to treat dysentery, cholera, typhoid fever, and plague in the pre-antibiotic era [7,8,9,10]. In the 1920s and 1930s, phage therapy was practiced in both Europe and the United States, with commercial phage preparations produced by companies such as Eli Lilly and Squibb [11]. However, early phage therapy suffered from inconsistent clinical outcomes, poorly standardized production protocols, a limited understanding of phage biology, and the inability to purify phages from bacterial debris, which often caused adverse reactions. With the mass production of broad-spectrum antibiotics in the 1940s, which offered reliable, broad-spectrum activity against most bacterial pathogens, medical researchers largely abandoned phage research [12]. Nevertheless, phage therapy is still in the process of development. Many new phages are being screened or modified, and many of them have undergone partial clinical trials [13].

Unlike broad-spectrum antibiotics that can disrupt the symbiotic microbiome and cause widespread drug resistance, phages show exquisite host specificity. They only target chosen pathogenic bacteria, while not affecting cells of other species or strains. In addition, phages self-replicate at the infected site, so as to achieve low-dose and efficient treatment. Despite these advantages, natural phages still have limitations that hinder their extensive clinical transformation; they include narrow host spectrum, rapid clearance by the human immune system, limited biofilm penetration, and the potential to carry virulent or antibiotic-resistant genes.

To address these evolutionary constraints, the field of phage therapy has undergone a paradigm shift from passive isolation of natural phages to rational design of engineered phages. Advances in synthetic biology, particularly CRISPR-Cas genome editing, high-throughput sequencing, and structural biology, have enabled precise modification of phage genomes to enhance their therapeutic properties. This review provides a comprehensive analysis of the core advantages and limitations of phage therapy, details the synthetic biology toolkits driving phage engineering, and presents an in-depth examination of recent clinical applications and engineering breakthroughs. Finally, it discusses the remaining challenges to clinical translation and outlines future prospects, with a concise overview of how artificial intelligence (AI) may accelerate the next generation of phage therapeutics.

## 2. Core Advantages and Natural Limitations of Phage Therapy

### 2.1. Unique Biological Advantages of Phage Therapy

**Figure 1:**
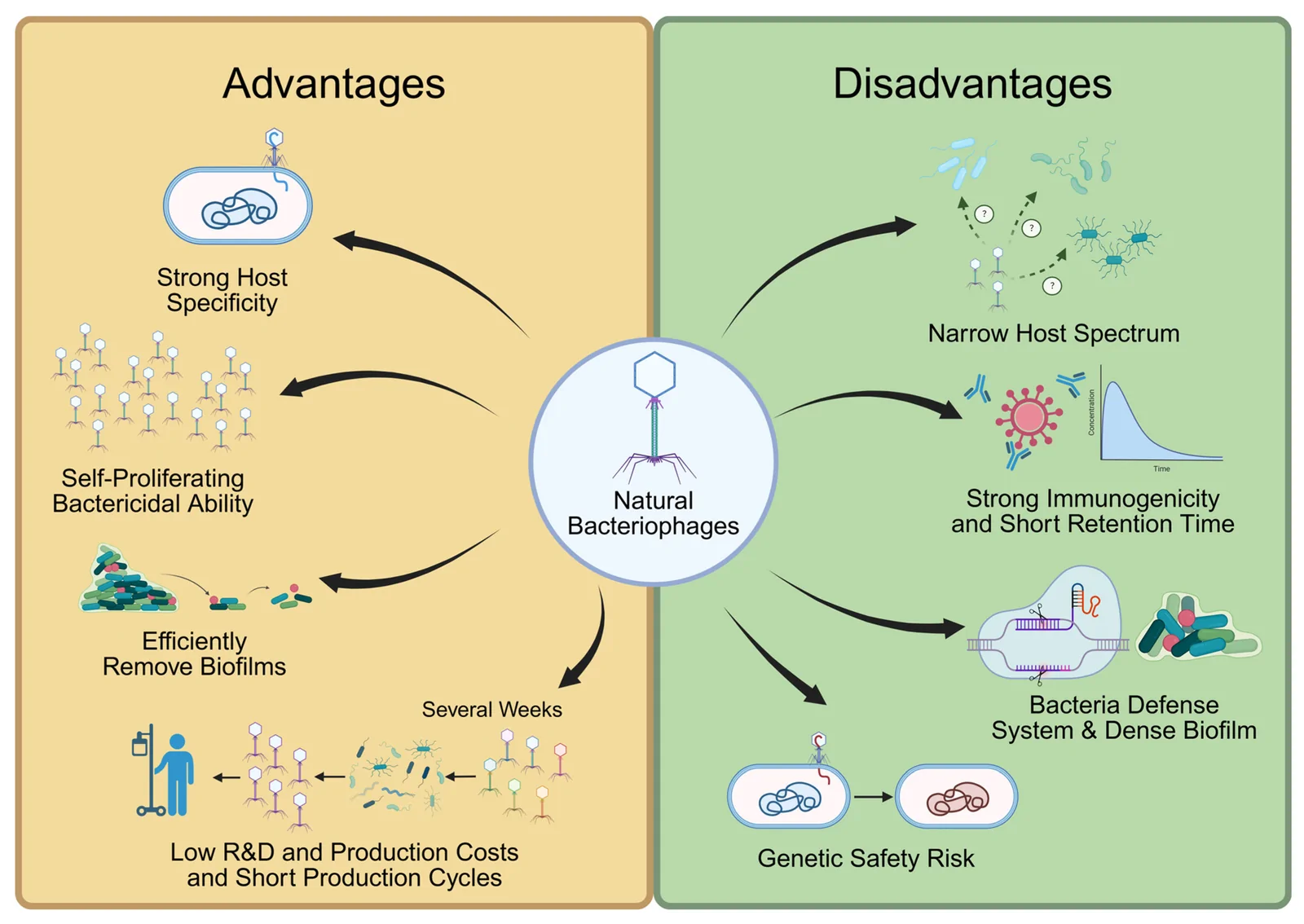
Core advantages and limitations of natural bacteriophages for therapeutic applications. Natural bacteriophages possess several inherent biological strengths that make them attractive antibacterial agents, including strong host specificity, minimizing disruption to the commensal microbiome, self-proliferating bactericidal activity that enables low-dose efficacy, efficient biofilm degradation capability via polysaccharide depolymerases, and low R&D/production costs with short development cycles (typically completed in several weeks). However, they also face critical clinical bottlenecks: narrow host spectrum limits broad applicability and requires personalized cocktails; strong immunogenicity and rapid systemic clearance (short serum half-life) reduce in vivo persistence; bacterial defense mechanisms (e.g., CRISPR-Cas systems) and dense biofilm matrices can block phage infection; and genetic safety risks (e.g., lysogenic conversion and generalized transduction) raise regulatory concerns. Created in BioRender. ZHOU, H. (2026) https://BioRender.com/0bcizv5 (accessed on 18 June 2026).

Bacteriophages are the most abundant biological entities on Earth, with an estimated 10^31^ phage particles in the biosphere, outnumbering bacteria tenfold. They are obligate intracellular parasites that rely entirely on bacterial metabolic machinery for replication, and this specialized lifestyle endows phage therapy with several unique advantages over conventional antibiotics (Figure 1).

First, exquisite host specificity is the defining feature of phages. Each phage recognizes and infects only a narrow range of bacterial strains, typically within a single species, via highly specific interactions between its surface receptor-binding proteins (RBPs) and complementary receptors on the bacterial surface. These bacterial receptors include lipopolysaccharides (LPS) in Gram-negative bacteria, peptidoglycans in Gram-positive bacteria, capsular polysaccharides, outer membrane proteins, flagella, and pili. This precision targeting avoids collateral damage to the human commensal microbiome caused by broad-spectrum antibiotics, which can disrupt the delicate balance of the gut, skin, and respiratory microbiomes [14,15].

Numerous clinical studies have demonstrated that phage therapy has minimal impact on the commensal microbiome. Volker Mai et al. demonstrated that the bacteriophage cocktail ShigActive™ significantly reduces *Shigella* colonization and shedding in mice without adversely affecting the gut microbiota, thus holding substantial potential as a therapeutic intervention. In this study, the researchers employed a C57BL/6J mouse model: following oral challenge with a susceptible *Shigella* strain, mice were administered either the ShigActive™ phage cocktail or ampicillin for treatment. The results revealed that ShigActive™ reduced the colony-forming unit (CFU) counts of the challenge strain by 10- to 100-fold in both feces and cecal contents, exhibiting therapeutic efficacy comparable to that of ampicillin while exerting a markedly smaller impact on the gut microbiota. Long-term safety studies further confirmed that phage therapy was well-tolerated with no detectable adverse effects and did not induce significant alterations in the composition of the gut microbiota [16]. This preservation of the commensal microbiome lowers the likelihood of antibiotic-associated diarrhea and helps prevent long-term disruption of microbial balance, a factor increasingly associated with conditions such as obesity, diabetes, inflammatory bowel disease, and certain mental health disorders. Furthermore, phage specificity minimizes the selective pressure for resistance development in non-target bacteria, limiting the spread of resistance genes across microbial communities [17].

Second, self-amplifying bactericidal activity enables phages to exert therapeutic effects at extremely low doses. Lytic phages follow a well-characterized replication cycle consisting of five stages: adsorption, penetration, biosynthesis, assembly, and release. Upon adsorption to a specific bacterial receptor, the phage injects its genetic material into the bacterial cell. The phage genome then hijacks the bacterial metabolic machinery to produce hundreds of copies of phage proteins and nucleic acids, which assemble into progeny phage particles. Finally, the phage produces lytic enzymes that degrade the bacterial cell wall, causing the cell to burst and release 50–200 progeny virions into the surrounding environment. These progeny phages then go on to infect neighboring bacteria, resulting in an exponential amplification of phage numbers at the site of infection [18,19].

This multi-hit effect means that phage concentrations increase in proportion to bacterial load, ensuring that even low initial doses of phages can effectively eliminate large bacterial populations. Once the bacterial population is eradicated, there are no longer any host cells for phage replication, and phages are rapidly cleared by the host immune system, leaving no residual drug in the body. This is in stark contrast to antibiotics, which require repeated administration to maintain therapeutic concentrations and can accumulate in the body, causing toxic side effects [20].

Third, biofilm clearance capability addresses a major unmet clinical need. Approximately 80% of chronic bacterial infections are associated with biofilms. Biofilms are structured, multicellular communities of bacteria embedded in a self-produced extracellular polymeric substance (EPS) matrix composed of polysaccharides, proteins, nucleic acids, and lipids. Biofilms confer up to 1000-fold increased resistance to antibiotics through multiple mechanisms: the EPS matrix acts as a physical barrier that limits drug penetration; bacteria within biofilms exhibit a gradient of metabolic activity, with cells in the inner layers entering a dormant, non-replicating state that is tolerant to most antibiotics; and biofilms promote horizontal gene transfer of resistance genes between bacterial cells [21].

In contrast, many phages produce polysaccharide depolymerases that specifically degrade the polysaccharide components of the biofilm matrix, exposing underlying bacteria to phage infection. Phages can also penetrate the biofilm matrix and infect bacteria within all layers of the biofilm, including dormant, metabolically inactive bacteria, which are largely unaffected by conventional antibiotics [22,23]. Hughes et al. isolated a bacteriophage designated SF153b that encodes a polysaccharide depolymerase with specific degradative activity against the extracellular polysaccharide (EPS) produced by *Staphylococcus aureus* strain 53b. Their experiments demonstrated that phage-mediated biofilm disruption occurs through a dual mechanism: the phage-encoded depolymerase enzymatically degrades the structural EPS matrix, while the phage simultaneously infects and lyses bacteria embedded within the inner layers of the biofilm. Scanning electron microscopy (SEM) imaging provided direct visual evidence that this specific phage caused extensive, large-scale degradation of susceptible biofilm structures [24]. In a separate study, Wu et al. isolated bacteriophage SH-KP152226 from clinical samples of multidrug-resistant *Klebsiella pneumoniae* and identified a novel polysaccharide depolymerase, Dep42, encoded by its tail fiber protein. Functional characterization confirmed that Dep42 exhibits strict specificity for K47 capsular polysaccharide. Notably, Dep42 not only significantly inhibited the initial formation of *Klebsiella pneumoniae* biofilms but also effectively degraded pre-formed, mature biofilms that are notoriously recalcitrant to antibiotic treatment [25]. Collectively, these mechanistic studies provide direct experimental validation of the dual-action biofilm clearance pathway of depolymerase-producing phages, highlighting the critical role of phage-encoded polysaccharide depolymerases in overcoming the biofilm-associated antibiotic resistance barrier.

Finally, phages offer rapid development timelines and low production costs. Unlike small-molecule antibiotics, which require an average of 10–15 years and high cost in research and development investment, phages can be readily recovered from diverse environmental reservoirs such as wastewater, soil, aquatic systems, and animal excreta, and subsequently characterized within a matter of weeks. Large-scale production of phages is relatively inexpensive, as they can be propagated in bacterial cultures using standard bioreactor technology. This makes phages particularly suitable for low-resource settings where access to expensive antibiotics is limited, and for rapid response to emerging AMR outbreaks [26,27].

### 2.2. Clinical Application Bottlenecks of Natural Phages

Despite their therapeutic promise, natural phages possess several inherent limitations that have impeded their widespread clinical adoption. Narrow host range is the most significant barrier to broad clinical use. Most natural phages infect only a small subset of strains within a bacterial species, while clinical isolates exhibit extensive genetic and phenotypic diversity [28]. For example, *Staphylococcus aureus* has over 20 different clonal complexes, each with unique surface receptors that are recognized by different phages [29]. This narrow host range necessitates the development of personalized phage cocktails for each patient, a process that is currently time-consuming and labor-intensive, often taking weeks to complete, but it is too slow for critically ill patients with rapidly progressing infections [30].

Immunogenicity and rapid systemic clearance limit the efficacy of intravenously administered phages. As foreign viral particles, phages are rapidly recognized by the innate immune system upon entering the bloodstream. The complement system is activated, leading to opsonization of phage particles by complement proteins, which are then recognized and phagocytosed by macrophages and neutrophils in the liver, spleen, and lungs [31]. Intravenous phages typically have a serum half-life of only 15–60 min, making it difficult to maintain therapeutic concentrations in the bloodstream for extended periods [32].

Additionally, repeated phage administration can induce the adaptive immune system to produce neutralizing antibodies, which bind to phage particles and prevent them from infecting bacteria. Maciej Zaczek et al. quantified the serum levels of IgG, IgA, and IgM anti-phage antibodies in 20 patients undergoing phage therapy from the Phage Therapy Center of the Hirszfeld Institute in Poland and systematically assessed the neutralizing activity of these antibodies. The patients received phage cocktails via oral and/or topical administration routes, and the core findings demonstrated that the majority of patients did not develop significantly elevated anti-phage antibody titers. Even in the subset of patients who mounted an antibody response, which was predominantly mediated by IgG and IgM, the emergence of anti-phage antibodies did not directly correlate with clinical treatment failure. Paradoxically, unfavorable therapeutic outcomes were instead observed in some patients with blunted antibody responses. This clinical investigation highlights that the relationship between neutralizing antibodies and clinical efficacy may be substantially more complex than previously hypothesized [33]. It is important to note that some studies pointed out that local administration of phages (e.g., topical application to wounds, aerosol inhalation into the lungs, or instillation into the urinary tract) is associated with much lower immunogenicity, as the local immune response is less robust than the systemic immune response [34].

Variable biofilm degradation activity remains a challenge. While some phages produce potent polysaccharide depolymerases that effectively degrade biofilm matrices, many natural phages lack this ability or produce depolymerases with narrow substrate specificity [35]. Mature biofilms with complex, multilayered structures are particularly resistant to phage infection, as phages cannot penetrate deeply into the dense EPS matrix to reach inner bacterial cells [36]. Furthermore, bacteria within biofilms can upregulate defense mechanisms such as CRISPR-Cas systems and restriction-modification systems, which protect them from phage infection [37].

Genetic safety risks are a major regulatory concern. Lysogenic (temperate) phages integrate their genomes into the bacterial chromosome as prophages, which can replicate along with the bacterial genome and be passed on to daughter cells. In the lysogenic state, prophages can carry and transfer virulence genes (e.g., genes encoding botulinum toxin, diphtheria toxin, or Shiga toxin) or antibiotic resistance genes via horizontal gene transfer [38]. Even lytic phages may occasionally package bacterial DNA during replication, a process known as generalized transduction, which can facilitate the spread of resistance genes between bacterial strains. Junxuan Zhang et al. isolated a T4-like lytic bacteriophage designated AH67C600_Q9, and demonstrated that this phage can mispackage host plasmid DNA and randomly incorporate fragments of the bacterial genomic DNA into its capsid during progeny virion assembly [39]. These safety concerns have led to strict regulatory requirements for phage therapeutics, particularly for engineered phages, and have slowed the clinical translation of phage therapy in many countries.

## 3. Synthetic Biology-Driven Phage Engineering Technology Foundation

**Figure 2:**
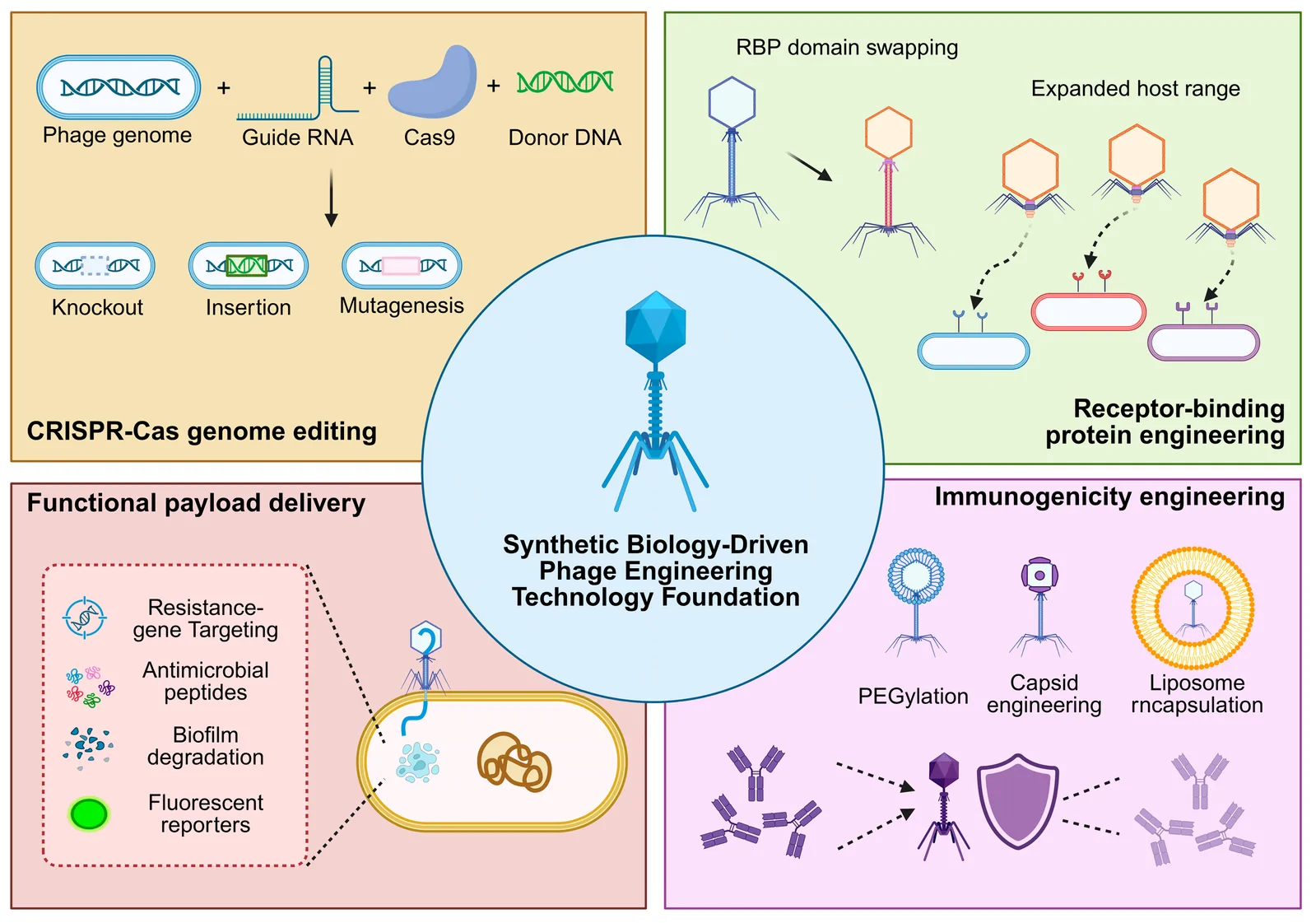
Synthetic biology-driven technology platforms for phage engineering. Synthetic biology provides four complementary engineering routes for improving phage therapeutics. CRISPR-Cas genome editing enables precise gene knockout, insertion, and mutagenesis; receptor-binding protein (RBP) engineering redirects or expands host range through modular domain swapping; functional payload delivery equips phages with resistance-gene-targeting systems, antimicrobial peptides, biofilm-degrading enzymes, or fluorescent reporters; and immunogenicity engineering, including PEGylation, capsid modification, and liposome encapsulation, aims to enhance stability, biocompatibility, and in vivo persistence. Created in BioRender. ZHOU, H. (2026) https://BioRender.com/cy5ighp (accessed on 5 August 2026).

Synthetic biology has revolutionized phage therapy by enabling precise, rational modification of phage genomes or phage virions to overcome their natural limitations (Figure 2). The development of CRISPR-Cas genome editing tools, in particular, has transformed phage engineering from a trial-and-error process to a systematic design discipline, allowing researchers to create engineered phages with enhanced therapeutic properties.

### 3.1. Genetic Engineering of Natural Phages

CRISPR-Cas genome editing is the most widely used tool for phage engineering. The CRISPR-Cas9 system, originally discovered as a bacterial adaptive immune system, allows for site-directed mutagenesis, gene knockout, and gene insertion in phage genomes with unprecedented precision and efficiency [40]. To edit a phage genome using CRISPR-Cas9, researchers design a guide RNA (gRNA) that targets a specific sequence in the phage genome. The Cas9 nuclease then cleaves the phage genome at the target site, creating a double-strand break. This break is repaired by homologous recombination with a donor DNA template that contains the desired genetic modification, resulting in a precisely edited phage genome. Marie-Laurence Lemay et al. achieved precise genetic modification of the genome of the virulent *Lactococcus lactis* bacteriophage p2 by employing the *Streptococcus pyogenes* CRISPR-Cas9 system expressed in a heterologous host [41].

CRISPR-Cas9 has been used to engineer phages for a variety of therapeutic purposes. For example, Joo Youn Park et al. knocked out the integrase and repressor genes of lysogenic phages to convert them into strictly lytic phages, eliminating the risk of lysogenic conversion and horizontal gene transfer. The insertion of heterologous genes encoding polysaccharide depolymerases, lysozymes, or antimicrobial peptides enhanced phage bactericidal activity and biofilm degradation capability [42]. In addition to Cas9, other CRISPR-Cas systems such as Cas12a, CasΦ, and Cas14 are also being used for phage engineering, offering advantages such as smaller size, higher specificity, and the ability to target multiple sites simultaneously [43,44].

Receptor-binding protein (RBP) engineering is the primary strategy for expanding phage host ranges. RBPs are the primary determinants of phage host specificity, and their modular structure makes them amenable to engineering. Most RBPs consist of two distinct domains: a C-terminal head domain that mediates binding to bacterial receptors, and an N-terminal tail domain that anchors the RBP to the phage tail. By swapping the head domains of RBPs between different phages, researchers can alter phage tropism to recognize new bacterial strains or serotypes [45,46].

Matthew Dunne et al. used *Listeria* phage PSA as a research model and identified Gp15 as the receptor-binding protein (RBP) of this phage. The research team constructed a synthetic phage library containing random-sequence RBP variants, screened for mutants with altered host ranges, and integrated these mutants to generate synthetic multivalent phages with expanded host tropism. The team further resolved the crystal structure of the carboxy-terminal domain of Gp15 responsible for receptor binding. Combined with bioinformatic approaches, they systematically identified compatible phage-encoded RBPs targeting different *Listeria* serotypes, enabling the rational design of chimeric RBPs. By swapping the head, neck, and shoulder domains of different RBPs, the researchers successfully generated chimeric phages with predictable and expandable host ranges [14].

Functional payload delivery expands the therapeutic potential of phages beyond their natural lytic activity. Phages can be engineered to deliver a variety of functional genes into bacterial cells, turning them into smart therapeutic agents that can perform specific tasks. One of the most promising applications of functional payload delivery is the use of phages to deliver CRISPR-Cas systems that specifically target and cleave antibiotic resistance genes. When delivered into a bacterial cell, the CRISPR-Cas system cuts the resistance gene, rendering the bacterium susceptible to conventional antibiotics. This approach has been successfully used to restore susceptibility to carbapenems, cephalosporins, and fluoroquinolones in multidrug-resistant bacteria [47,48,49].

Other payloads being explored include genes encoding antimicrobial peptides, which are small, positively charged peptides that disrupt bacterial cell membranes; quorum-sensing inhibitors, which block bacterial cell-to-cell communication and prevent biofilm formation; and fluorescent reporters such as green fluorescent protein (GFP) or luciferase, which enable real-time imaging of bacterial infections in vivo. Phages can also be engineered to deliver toxin genes, such as the gene encoding diphtheria toxin A chain, which kills bacterial cells by inhibiting protein synthesis [50,51,52,53].

Immunogenicity engineering aims to improve phage stability and circulation half-life in vivo. Chemical modification of phage capsids with polyethylene glycol (PEG) is the most widely used method to reduce phage immunogenicity. PEG is a hydrophilic polymer that forms a protective coating around the phage particle, shielding it from recognition by the immune system [31]. Md Shamsuzzaman et al. performed covalent PEGylation of four lytic *Escherichia coli* bacteriophages using 5 kDa methoxypolyethylene glycol succinimidyl ester (mPEG-S-NHS), achieving a surface amine modification rate of over 60% on the phage capsid. The experimental results demonstrated that PEGylation significantly enhanced the in vivo circulatory persistence, intracellular stability, and immunomodulatory activity of the phages. PEGylated phages exhibited markedly prolonged serum half-lives, reached higher plasma titers in infected hosts, showed reduced clearance rates, and accelerated bacterial eradication. Furthermore, PEGylated phages effectively suppressed infection-induced aberrant elevation of cytokines and restored the normal cytokine profile [54].

Genetic engineering approaches are also being explored to reduce phage immunogenicity. These include humanization of phage capsid proteins, which involves replacing immunodominant epitopes on the phage surface with human protein sequences; deletion of immunodominant epitopes from capsid proteins; and engineering phages with chimeric capsids that combine components from different phages to reduce antigenicity [55]. Another promising approach is the encapsulation of phages in liposomes or nanoparticles, which not only reduces immunogenicity but also protects phages from degradation by enzymes and pH changes in the body [56].

### 3.2. Non-Genetic Engineering Modifications of Phages

Beyond genetic editing, non-genetic physical and chemical strategies have become important for addressing pharmacokinetic and delivery limitations of natural phages without altering their genomes. These approaches broadly include formulation-based encapsulation or embedding, as well as covalent or non-covalent surface functionalization.

Liposomal encapsulation can protect bacteriophages from the impact of harsh gastrointestinal conditions, reduce the neutralization of antibodies and prolong systemic exposure according to the preparation and route. The basic formula work summarized by Malik and his colleagues established liposomes as a multifunctional protective matrix for bacteriophage delivery [56]. More recent charge-driven micromixing and microfluidic methods have achieved approximately 90–91% encapsulation efficiency for both short- and long-tailed phages while largely preserving viability [57].

Hydrogel-based carriers enable local, sustained phage release over days to weeks and are being explored for implant-associated infections and other localized applications. Thermo-responsive HA-pNIPAM hydrogels and alginate–chitosan microbeads support controlled local release whose kinetics depend on both the matrix and the phage morphotype [58]. At the same time, related alginate/chitosan-modified gelatin microbeads further illustrate tunable release and improved storage stability [59]. 3D-printable alginate–methylcellulose (AlgMC) inks release active phages for up to 35 days under near-physiological in vitro conditions, supporting their potential for local therapy of implant-associated infections [60].

Magnetic nanoparticle conjugation (e.g., Fe_3_O_4_–phage hybrids) enables external-field-guided enrichment and can improve localized antibacterial or antibiofilm activity in preclinical models. Magnetically controlled infection-site accumulation remains an emerging, largely early-stage therapeutic strategy [61].

Complementary surface chemistries (mainly NHS and EDC-mediated bioconjugation) can efficiently attach functional molecules such as polymers, photosensitizers or gold nanorods to bacteriophage housing. Among them, polyethylene glycol (PEG) modification can significantly reduce immunogenicity and improve pharmacokinetic behavior. The coupling of photosensitizers or plasma nanomaterials, however, gives bacteriophages photodynamic or photothermal sterilization ability to achieve multimodal antibacterial effects [62,63]. For example, thiolation-mediated conjugation of gold nanorods to phages yields photothermal “phanorod” constructs that selectively ablate target bacteria [64]. In murine wound-infection models, related phage–gold nanorod hybrids can further incorporate Zn^2+^ release to accelerate healing while limiting systemic toxicity [65].

Collectively, these non-genetic approaches show promise for improving in vivo stability, local delivery and therapeutic performance, and they may be combined with the genetic toolkits described above when compatibility with infectivity and host-range determinants is carefully evaluated.

## 4. Clinical Applications and Engineering Progress of Phage Therapy

### 4.1. Clinical Application Cases of Natural Phages

In recent years, phage therapy has transitioned from a niche experimental treatment to a clinically viable option for multidrug-resistant infections. While most clinical applications remain in the compassionate use setting, an increasing number of formal clinical trials are demonstrating the safety and efficacy of phage therapy, and regulatory agencies around the world are beginning to establish frameworks for the approval of phage therapeutics [66].

#### 4.1.1. A Landmark Case of Phage Therapy

The case of Tom Patterson remains the most famous example of successful phage therapy. Robert T. Schooley et al. reported an experimental phage therapy case involving a 68-year-old diabetic patient with necrotizing pancreatitis complicated by multidrug-resistant *Acinetobacter baumannii* infection. Despite treatment with all available antibiotics and percutaneous drainage, the patient’s condition continued to deteriorate for over 4 months, progressing to multiple organ failure. In the absence of effective antimicrobial options, researchers from the U.S. Naval Medical Research Center and the Phage Technology Center at Texas A&M University screened a global phage collection and identified 9 lytic phages with specific activity against the patient’s *A. baumannii* isolate. A personalized phage cocktail was administered via two complementary routes: intravenous infusion and percutaneous injection directly into the abscess cavities. Following phage administration, the patient’s downward clinical trajectory was rapidly reversed. The *A. baumannii* infection was completely eradicated, and the patient ultimately achieved full functional recovery. This landmark case represents the first successful treatment of a pan-drug-resistant bacterial infection via intravenous phage administration under emergency investigational new drug (eIND) authorization from the U.S. Food and Drug Administration (FDA).

Since then, hundreds of compassionate use cases have been reported worldwide, covering a wide range of infections caused by ESKAPE pathogens. Jean-Paul Pirnay et al. conducted a landmark multinational retrospective cohort study coordinated by the Belgian Phage Therapy Consortium, covering 35 hospitals in 29 cities across 12 countries and analyzing a total of 100 cases of personalized bacteriophage therapy (BT) for refractory bacterial infections. The study revealed that personalized BT achieved an overall clinical improvement rate of 77.2% and a bacterial eradication rate of 61.3%, with the most common treatment indications being lower respiratory tract infections, skin and soft tissue infections, and bone infections. Notably, the study confirmed the necessity of adjunctive antibiotic therapy: phage monotherapy without concomitant antibiotics reduced bacterial eradication rates by 70% (OR = 0.3), generally consistent with the finding that 90% (9/10) of phage-antibiotic combinations exhibited in vitro synergy. Furthermore, de novo in vivo phage resistance emerged in 43.8% (7/16) of evaluable cases during treatment, highlighting the importance of rational multi-phage cocktail design to delay resistance development. In terms of safety, the therapy demonstrated a favorable overall tolerability profile: a total of 15 adverse events were reported, of which only 7 were non-serious adverse drug reactions, with no treatment-related fatalities [67].

#### 4.1.2. Formal Clinical Trials

Mian Li Ooi et al. reported a human open-label clinical trial investigating the safety, tolerability, and preliminary efficacy of an escalating-dose regimen of the experimental phage cocktail AB-SA01 in patients with chronic rhinosinusitis caused by methicillin-resistant *Staphylococcus aureus* (MRSA) infection. The study enrolled 9 eligible patients aged 18–70 years with MRSA-positive chronic rhinosinusitis, who were assigned to sequential dose-escalation cohorts receiving twice-daily nasal irrigation with AB-SA01 at total daily doses of 3 × 10^8^ plaque-forming units (PFU), 3 × 10^8^ PFU, and 3 × 10^9^ PFU respectively. The results demonstrated that all 9 participants completed the full study protocol. Intranasal phage therapy was well tolerated overall, with no serious adverse events or treatment-related deaths reported throughout the trial period. Among the 9 individuals, 2 showed evidence of clinical and microbiological infection clearance [68].

Another pivotal clinical trial was conducted by Vance G. Fowler Jr. et al. on Exebacase, a bacteriophage-derived staphylolytic lysin that functions as a direct lytic agent against *Staphylococcus aureus*. The study employed a randomized controlled trial (RCT) design, in which 121 patients with *S. aureus* bloodstream infection (BSI) or endocarditis were randomly assigned to receive a single dose of Exebacase or placebo, with all patients concurrently receiving standard-of-care antibiotic therapy. The results showed that the combination of Exebacase and antibiotics increased the clinical response rate at day 14 by 42.8 percentage points in the methicillin-resistant *S. aureus* (MRSA) subgroup (74.1% vs. 31.3%). The incidence of adverse events was comparable between the two groups, and no hypersensitivity reactions to Exebacase were reported. The 30-day all-cause mortality was 9.7% in the Exebacase group versus 12.8% in the antibiotic-only group, with a statistically significant difference observed specifically in MRSA patients (3.7% vs. 25.0%). Among US MRSA patients, Exebacase treatment reduced hospital length of stay by 4 days and decreased the 30-day readmission rate by 48%. These findings provide robust evidence for the feasibility of Exebacase and direct-acting lysins as promising therapeutic agents [69].

Subsequent complementary studies have further corroborated that Exebacase exerts rapid bactericidal activity against *S. aureus*, exhibits an inherently low propensity for the development of bacterial resistance, and can suppress the emergence of resistance to conventional anti-staphylococcal antibiotics [70,71].

However, a more recent phase 3 clinical trial, which randomized a total of 259 patients, was terminated early for futility. In the MRSA subgroup, the day 14 clinical response rate was 50.0% (32/64) in patients receiving Exebacase plus antibiotics versus 60.6% (20/33) in those receiving antibiotics alone, with no statistically significant difference observed between the two groups. Post hoc analysis suggested that the relatively small sample size of the trial may have contributed to imbalances in multiple components of the composite day 14 clinical outcome measure. Nonetheless, despite its premature termination, this trial remains a meaningful milestone in the advancement of phage therapy and has provided valuable lessons to inform the design and execution of future clinical investigations in this field [72].

Currently, there are several active clinical trials of phage therapy worldwide, evaluating phage therapeutics for a variety of indications, including skin and soft tissue infections, urinary tract infections, pulmonary infections, sepsis, and prosthetic device-associated infections. These trials are expected to provide robust evidence for the safety and efficacy of phage therapy and pave the way for broader regulatory approval in the coming years [73,74].

### 4.2. Research and Development Progress of Engineered Phages

Synthetic biology has enabled the development of engineered phages with enhanced therapeutic properties, addressing many of the limitations of natural phages. Recent advances in phage engineering have focused on expanding host ranges, improving bactericidal activity, overcoming bacterial defense systems, and delivering functional payloads.

#### 4.2.1. Host Range Engineering

Host range engineering is the most active area of phage engineering research, with the goal of developing broad-spectrum phages that can cover the majority of clinical isolates of a given pathogen. Wang et al. constructed a library of 114 *Klebsiella pneumoniae* phages and performed large-scale phage-host interaction analysis on 238 clinical isolates collected from hospitals across China. Using clustering algorithms, they identified six distinct RBP clusters that determine phage tropism for different capsular serotypes. Using these newly identified RBPs, researchers successfully engineered bacteriophages by modifying their receptor-binding proteins (RBPs). This alteration remodeled and broadened the phages’ specificity toward host capsules, thereby enabling a tunable and versatile phage therapy strategy for combating *Klebsiella pneumoniae* infections [75].

Based on this discovery, Shisong Jing et al. retrieved 280 non-redundant Przondovirus RBP sequences and resolved over 50 discrete clusters. Functional screening extended the experimentally verified capsular locus (KL) targets of Przondovirus from 14 to 32. The conserved anchor motifs facilitated plug-and-play modular assembly of Prz_RBP1 and Prz_RBP2. Exogenous supplementation with Prz_RBP2 variants realized predictable host range expansion, which establishes a universal paradigm for precision phage therapy [76].

#### 4.2.2. Lysin Engineering

Lysins (phage-encoded peptidoglycan hydrolases) are potent bactericidal agents that lyse bacterial cells by degrading their cell walls. Unlike intact phages, lysins are not immunogenic and can kill bacteria rapidly, regardless of their metabolic state. They are particularly effective against Gram-positive bacteria, which have a thick peptidoglycan cell wall that is exposed to the external environment [77,78].

Yue Zhang et al. developed an integrated software package termed DeepLysin, which leverages artificial intelligence to identify novel antibacterial phage lysins from the uncharacterized “dark matter” of phage genomes. In that study, they screened unannotated staphylococcal phage genomes and computationally predicted putative lysins. Seventeen newly identified lysins were randomly selected for experimental validation, among which seven exhibited potent antibacterial activity in vitro. Notably, LLysSA9 outperformed the currently most effective counterparts. The therapeutic efficacy of LLysSA9 was further validated in mouse models of bacteremia and wound infection. This work demonstrates the great potential of combining computational and experimental strategies to accelerate the discovery of uncharacterized antibacterial proteins encoded by phage genomes, offering a promising solution to the growing threat of antimicrobial resistance [79].

#### 4.2.3. Overcoming Bacterial Defense Systems

Bacteria have evolved a variety of sophisticated defense systems to resist phage infection, including CRISPR-Cas systems, restriction-modification systems, abortive infection systems, and superinfection exclusion systems. These defense systems can significantly reduce the efficacy of phage therapy by preventing phage adsorption, degrading phage DNA, or killing infected bacterial cells before progeny phages can be produced.

To overcome these defenses, researchers have engineered phages to express anti-defense proteins that inhibit bacterial immune systems. Erez Yirmiya et al. established a systematic pipeline to screen immune suppressors encoded by viral proteins. Using AlphaFold2-Multimer for co-folding analysis, this study focused on two major bacterial defense systems, Thoeris and CBASS (Cyclic Bacteriophage Anti-phage Signaling System), which are evolutionary ancestors of eukaryotic TIR (Toll/Interleukin-1 Receptor) and cGAS-mediated (cyclic GMP-AMP Synthase-mediated) immunity, respectively. Seven families of inhibitory proteins targeting Thoeris and CBASS were identified, corresponding to thousands of genes widely distributed across phage genomes. All validated suppressors were found to block the active sites of immune proteins via physical interaction. Introduction of these anti-defense genes enhanced phage infectivity by up to 1000-fold. Remarkably, phage-derived TIR suppressors were capable of inhibiting human and plant TIR immune proteins, while phage-encoded cGAS inhibitors also suppressed human cGAS. These findings demonstrate that phages constitute a rich reservoir of cross-species immune modulators [80].

In a separate study, Ana Rita Costa et al. experimentally characterized the anti-defense strategies employed by phages. Using Pseudomonas model phages as research objects, the team revealed that a single representative Pbunavirus encodes as many as five known anti-defense genes. These include TadIII-1 that targets Type III Thoeris, ZadI-1 against Type I Zorya, as well as the broad-spectrum inhibitors Bdi1 and Bdi2, which are able to repress four distinct defense systems simultaneously. This work indicates that phages have evolved both specialized and universal anti-defense mechanisms, providing direct guidance for improving the efficacy of phage therapy [81].

#### 4.2.4. Functional Payload Delivery

Engineered phages can be used as delivery vehicles for a variety of therapeutic payloads, expanding their utility beyond direct bacterial lysis. One of the most promising applications is the use of phages to deliver CRISPR-Cas systems that specifically target and cleave antibiotic resistance genes. This approach not only kills bacteria but also eliminates the resistance genes, preventing their spread to other bacteria.

Tomofumi Kawaguchi et al. engineered non-replicating phage capsids (designated antibacterial capsids, AB-Capsids) and packaged them with plasmids harboring the codon-optimized sequence of *Sphingobacterium spiritivorum* Cas13a (cas13aPA) and blaIMP-1-targeting guide RNA (gRNA), generating a novel antimicrobial construct. This construct specifically inhibited the growth of blaIMP-1-expressing *Pseudomonas aeruginosa* and significantly reduced the minimum inhibitory concentration (MIC) of imipenem. It exhibited bactericidal activity exclusively in the presence of the target gene or cognate gRNA, with no detectable off-target effects observed. Furthermore, the spacer-dependent and expression level-dependent characteristics of its bactericidal activity were rigorously validated using an inducible blaIMP-1 expression system. These findings demonstrate that AB-Capsids for programmable Cas13a delivery represent a gene-specific, non-replicating antimicrobial platform that effectively reverses bacterial antibiotic resistance, establishing a universal class of CRISPR-based antibiotic adjuvants [82].

Other payloads being explored further expand the functional versatility of engineered phages. Antimicrobial peptides (AMPs) can be delivered as genetic cargo to disrupt bacterial membranes and synergize with phage-mediated lysis, thereby enhancing overall bactericidal potency and reducing the likelihood of resistance emergence [83]. Biofilm-degrading enzymes, particularly polysaccharide depolymerases and DNases, enable phages to penetrate the extracellular polymeric matrix that protects biofilm-embedded cells. When expressed from engineered phages or co-administered as purified enzymes, these activities markedly improve access to otherwise refractory bacterial populations [25]. Fluorescent reporter genes (e.g., GFP or luciferase) allow real-time visualization of both bacterial infection dynamics and phage replication in vivo, converting phages into simultaneous therapeutic and diagnostic tools [51,52].

### 4.3. Practice of Personalized Phage Therapy

Phage therapy is inherently personalized, as the effectiveness of a phage cocktail depends on the specific bacterial strain infecting the patient. The standard workflow for personalized phage therapy involves five key steps: (1) isolation and identification of the pathogenic bacterial strain from the patient; (2) screening of a phage library to identify phages that lyse the patient’s strain; (3) formulation of a personalized phage cocktail containing 3–5 phages with different mechanisms of action; (4) in vitro susceptibility testing to confirm the efficacy of the cocktail; and (5) administration of the cocktail to the patient via the appropriate route.

To address the time constraints of this workflow, several institutions have developed rapid phage screening platforms that can identify effective phages within 24–48 h. Qi Wang et al. developed a nanodroplet dilution SlipChip (nd-SlipChip), which enables a three-tiered sequential therapeutic selection process encompassing antibiotic minimum inhibitory concentration (MIC) determination, antibiotic combination regimen evaluation, and phage screening, all completed within 2 h. This platform generates serial dilutions of antibiotics or phages via a microdroplet array and performs real-time monitoring of bacterial growth curves. Its efficacy was validated using 24 clinical isolates, including *E. coli*, *K. pneumoniae*, *A. baumannii*, and *S. aureus* [84].

In another parallel study, Jun Luo et al. took advantage of the basic characteristic that phages replicated a large number of intracellular copies after infecting live bacteria. Combined with TaqMan quantitative PCR (qPCR) technology to accurately quantify bacteriophage DNA amplification, this method can detect viable *A. baumannii* with a concentration as low as 50 CFU/mL within 3 h. In addition, they completed a broad-spectrum antibiotic sensitivity test within 2 h, and it only took 30 min of antibiotic exposure to obtain readable results. The verification results of 12 clinical isolates show that the method is completely consistent with the broth micro-dilution method as the “gold standard” (the compliance rate reaches 100%). With the technical breakthrough of each operation step, the development of the rapid bacteriophage screening platform has become more and more mature and stable [85,86,87].

Phage cocktail design is a key component of personalized treatment, as it can minimize the risk of bacterial drug resistance. The best mixture contains phages targeting different bacterial receptors and having non-overlapping drug resistance mechanisms. This ensures that if the bacteria are resistant to a phage in the mixture, it is still susceptible to the influence of other bacteriophages. Recent studies show that in most cases, a mixture containing 3 bacteriophages is enough to prevent the development of drug resistance, while a larger mixture will not provide additional benefits and may increase the risk of immune response [88,89].

The choice of administration route is also important for the success of phage therapy. Local administration (e.g., topical application to wounds, aerosol inhalation into the lungs, instillation into the urinary tract, or injection into infected joints) is generally preferred, as it delivers high concentrations of phages directly to the site of infection and minimizes systemic exposure, reducing the risk of immune reactions. Systemic administration (intravenous injection) is reserved for patients with disseminated infections such as sepsis, and requires careful monitoring of phage concentrations and immune responses [31,90].

## 5. Core Challenges Facing the Clinical Translation of Phage Therapy

**Figure 3:**
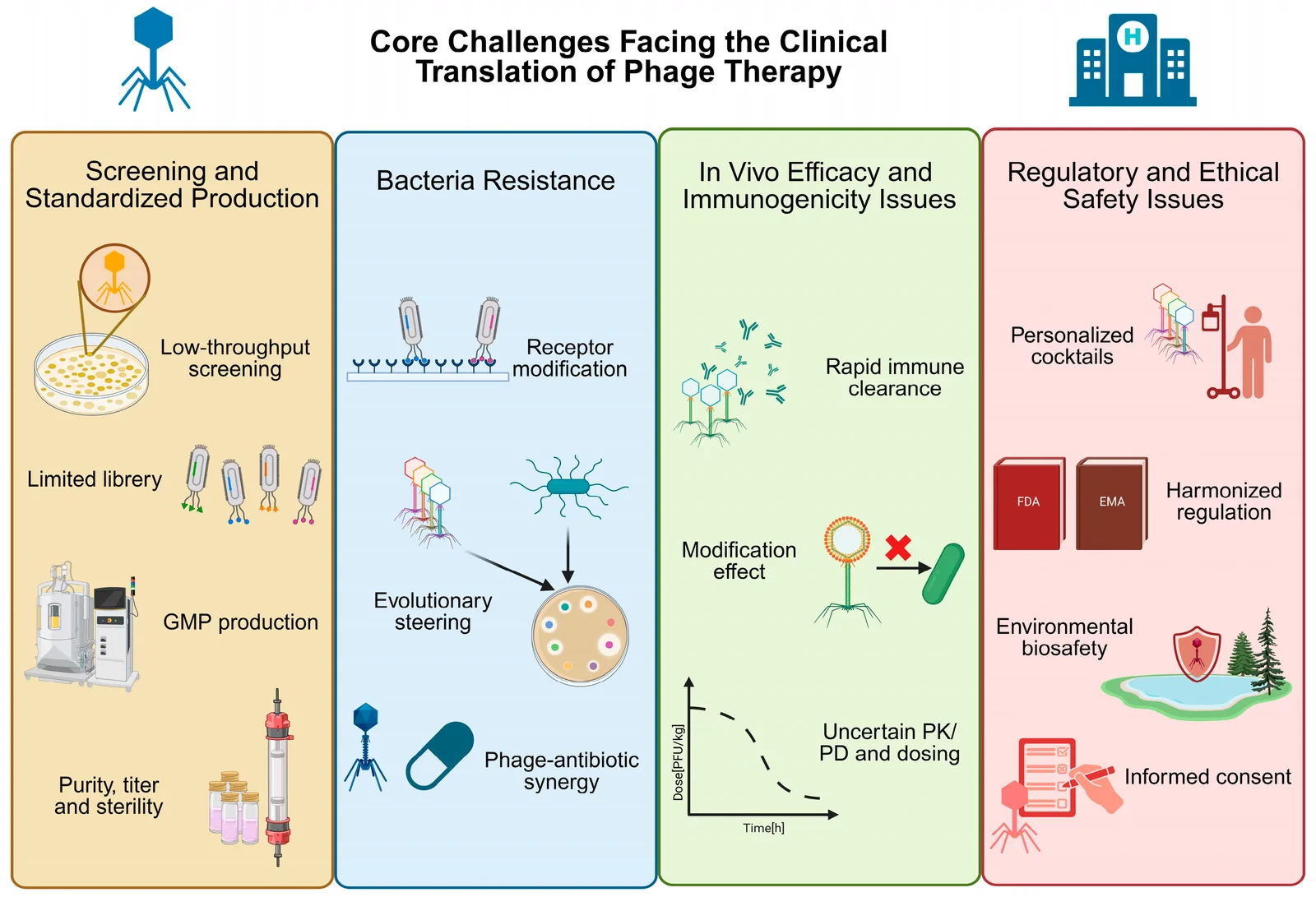
Core challenges facing the clinical translation of phage therapy. The principal barriers to routine clinical use span four interconnected domains: screening and standardized production, including low-throughput screening, limited phage libraries, GMP manufacturing, and quality control; bacterial resistance driven by receptor modification and evolutionary adaptation, which may be addressed through evolutionary steering and phage-antibiotic synergy; in vivo efficacy and immunogenicity, including rapid immune clearance, modification-dependent performance, and uncertain pharmacokinetics, pharmacodynamics, and dosing; and regulatory and ethical safety issues involving personalized cocktails, harmonized oversight, environmental biosafety, and informed consent. Created in BioRender. ZHOU, H. (2026) https://BioRender.com/0bcizv5 (accessed on 5 August 2026).

Despite the significant progress made in phage therapy research, several major challenges remain to be addressed before it can become a mainstream clinical treatment (Figure 3).

### 5.1. Phage Screening and Standardized Production Issues

Current phage screening methods are still largely manual and low-throughput, making it difficult to rapidly identify effective phages for emerging resistant strains. While high-throughput screening platforms are being developed, they are expensive and not widely available, particularly in low-resource settings. Additionally, most phage libraries are small and limited to a few common pathogens, meaning that effective phages may not be available for rare or emerging resistant strains.

Another major challenge is the lack of standardized protocols for phage production, purification, and quality control. Phage products produced by different laboratories vary widely in purity, potency, and safety, which complicates clinical trial design and regulatory approval. Traditional phage production methods involve propagating phages in bacterial cultures, followed by purification using centrifugation and filtration. However, these methods often result in phage preparations that contain residual bacterial debris, endotoxins, and other contaminants, which can cause adverse reactions in patients.

The development of standardized, scalable production processes is essential for the commercialization of phage therapeutics. This includes the development of good manufacturing practice (GMP)-compliant production facilities, standardized purification methods such as chromatography, and validated quality control assays for measuring phage titer, purity, endotoxin content, and sterility.

### 5.2. Bacterial Resistance Issue

While phage cocktails reduce the risk of resistance development, bacteria can still evolve resistance to phages through multiple mechanisms. These include mutations in phage receptors that prevent phage adsorption, downregulation of receptor expression, activation of CRISPR-Cas or restriction-modification systems that degrade phage DNA, and abortive infection systems that kill infected bacterial cells before progeny phages can be produced. Bacteria can also develop resistance to multiple phages simultaneously, making phage therapy ineffective [91].

Understanding the molecular mechanisms of phage resistance and developing strategies to overcome it is a major research priority. One promising approach is the use of “evolutionary steering,” where phages are pre-adapted to bacterial resistance mutations in the laboratory [92]. By serial passaging phages on resistant bacterial strains, researchers can select phage mutants that have regained the ability to infect the resistant bacteria. This approach has been successfully used to generate phages that can infect resistant strains of *Staphylococcus aureus* [93].

Another approach is the use of phage-antibiotic combination therapy, which can prevent the development of both phage resistance and antibiotic resistance [94]. Many antibiotics and phages exhibit synergistic activity, meaning that their combined effect is greater than the sum of their individual effects. For example, antibiotics that damage the bacterial cell wall can increase phage adsorption and penetration, while phages can disrupt biofilms, making bacteria more susceptible to antibiotics. Additionally, the use of multiple antibiotics and phages with different mechanisms of action makes it much more difficult for bacteria to develop resistance [95].

### 5.3. In Vivo Efficacy and Immunogenicity Issues

The efficacy of systemically administered phages remains limited by their rapid clearance by the immune system. While PEGylation and other chemical modifications can extend phage circulation half-life, these modifications may also reduce phage infectivity by blocking the RBP or altering the phage capsid structure. Developing genetically engineered phages with reduced immunogenicity and improved tissue penetration is an active area of research.

Additionally, the pharmacokinetics and pharmacodynamics of phages in humans are still poorly understood. Phage pharmacokinetics are complex and depend on multiple factors, including the phage strain, administration route, patient immune status, and the site of infection. There is currently no standardized dosing regimen for phage therapy, and dosing is often based on empirical evidence rather than pharmacokinetic data. More studies are needed to characterize the pharmacokinetics and pharmacodynamics of phages in humans, and to develop optimized dosing regimens and administration routes.

### 5.4. Regulatory and Ethical Safety Issues

Phages are living biological agents, and their regulatory classification differs from traditional small-molecule drugs and protein therapeutics. Current regulatory frameworks are not well-suited to address the unique challenges of phage therapy, particularly personalized phage cocktails, which are custom-made for individual patients and cannot be tested in large-scale clinical trials. There is an urgent need for the development of clear, harmonized regulatory guidelines for phage therapeutics that balance safety and innovation.

In recent years, several countries have taken steps to establish regulatory frameworks for phage therapy. The United States FDA has issued guidance on the development of phage therapeutics, and allows for the use of phage therapy under the compassionate use program and the investigational new drug (IND) pathway. The European Medicines Agency (EMA) has also issued guidance on the development of advanced therapy medicinal products (ATMPs), which includes phage therapeutics [96,97,98].

Ethical issues also need to be addressed, including the environmental release of engineered phages and the informed consent of patients. Engineered phages could potentially spread into the environment and have unintended ecological consequences, such as disrupting natural microbial communities or transferring engineered genes to other organisms. Rigorous risk assessment is required to ensure the safe use of engineered phages, and measures should be taken to prevent their release into the environment. Additionally, since phage therapy is still an experimental treatment in many countries, patients must be fully informed of the risks and benefits before giving their consent.

## 6. Future Development Directions and Prospects

**Figure 4:**
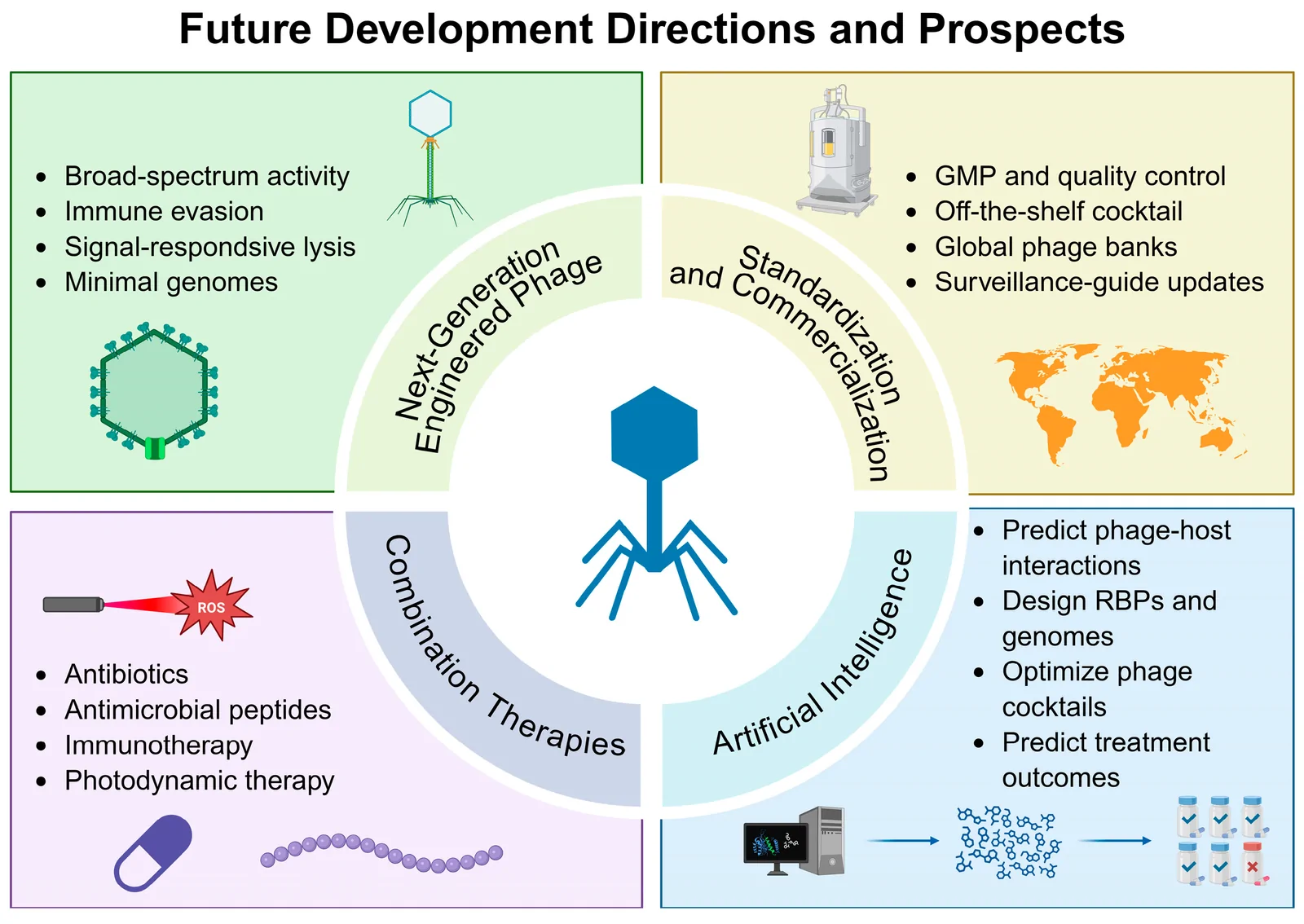
Future development directions and prospects for phage therapy. Future progress is expected to converge along four complementary directions: next-generation engineered phages with broader host range, immune-evasion properties, signal-responsive lysis, and minimized genomes; standardization and commercialization through GMP manufacturing, off-the-shelf cocktails, global phage banks, and surveillance-guided updates; artificial intelligence for predicting phage-host interactions, designing receptor-binding proteins and genomes, optimizing phage cocktails, and forecasting treatment outcomes; and combination therapies integrating phages with antibiotics, antimicrobial peptides, immunotherapy, or photodynamic therapy. Created in BioRender. ZHOU, H. (2026) https://BioRender.com/07lcbx6 (accessed on 5 August 2026).

Phage therapy is poised to become an essential component of the global armamentarium against multidrug-resistant bacterial infections. While significant challenges remain, advances in synthetic biology, microbiology, and clinical research are rapidly addressing these limitations (Figure 4).

### 6.1. Next-Generation Engineered Phages

Future phage engineering efforts will focus on developing “smart” phages with enhanced therapeutic properties. These include phages with broad-spectrum activity against multiple bacterial species, phages that can evade the human immune system, and phages that can sense and respond to specific bacterial signals (e.g., quorum-sensing molecules) [99]. For example, researchers are developing “quorum-sensing phages” that only express lytic genes when they detect high concentrations of bacterial quorum-sensing molecules, ensuring that they only replicate and lyse bacteria at the site of infection, minimizing collateral damage to the commensal microbiome [100,101].

The development of minimal phage genomes, which contain only the essential genes required for replication and infection, will improve the safety and controllability of engineered phages. Minimal phage genomes are smaller and easier to manipulate than natural phage genomes, and they eliminate the risk of carrying virulence or antibiotic resistance genes. Additionally, minimal phages can be engineered to carry larger therapeutic payloads, expanding their functional capabilities [43,102].

### 6.2. Standardization and Commercialization

The establishment of standardized production protocols, quality control assays, and regulatory guidelines will be critical for the commercialization of phage therapeutics. The development of off-the-shelf phage cocktails that cover the most common multidrug-resistant pathogens will make phage therapy more widely accessible and reduce the need for personalized treatment. These off-the-shelf cocktails can be formulated based on epidemiological data to cover the most prevalent resistant strains in a given region, and will be updated regularly to reflect changes in resistance patterns.

Additionally, the establishment of global phage banks and sharing networks will facilitate the rapid identification and distribution of effective phages for emerging infections. Through large-scale collection, characterization and indexing of bacteriophages, this bank will provide researchers and clinicians with access to a diverse collection of phages, enabling rapid response to AMR outbreaks [103].

### 6.3. Combination Therapies

Phage therapy is most effective when used in combination with other antimicrobial agents. Future research will focus on identifying synergistic combinations of phages and antibiotics, immunotherapies, or antimicrobial peptides that enhance bactericidal activity and prevent resistance development. For example, phages may be used in combination with immune checkpoint inhibitors to enhance the host immune response to bacterial infections. Phage infection can activate the innate immune system, and immune checkpoint inhibitors can prevent the immune system from being suppressed by bacteria, resulting in a synergistic effect that enhances bacterial clearance [104,105].

Another promising combination is phage therapy and photodynamic therapy, which uses light-activated compounds to generate reactive oxygen species that kill bacteria. Phages can be engineered to target bacteria specifically, and photodynamic therapy can then be used to kill the phage-infected bacteria, resulting in a highly effective and targeted treatment [106,107,108].

### 6.4. Role of Artificial Intelligence

Artificial intelligence will play an increasingly important role in accelerating phage therapy development. Machine learning models can predict phage-host interactions from genomic data, enabling rapid identification of effective phages for specific bacterial strains. Generative AI can design novel RBPs with expanded host ranges and engineer phage genomes with enhanced therapeutic properties. Additionally, AI can optimize phage cocktail design and predict treatment outcomes, improving the efficacy of personalized phage therapy.

## 7. Conclusions

Phage therapy provides a promising solution to the global antibacterial drug resistance crisis. With its unique mechanism of action, high specificity and low toxicity, bacteriophage provides a complementary means to traditional antibiotics for the treatment of multidrug-resistant bacterial infections. The progress of synthetic biology has enabled people to rationally design engineered phages with enhanced therapeutic properties, so as to overcome many limitations of natural phages. Although there are still serious challenges in production standardization, regulatory approval and in vivo efficacy, the rapid progress of phage research in the past ten years shows that these problems are expected to be solved in the next few years. As phage therapy moves from laboratory to clinical application, it is tempting to speculate that it may change the treatment mode of bacterial infections.

## Data Availability

No new data were created or analyzed in this study. Data sharing is not applicable to this article.
